# Effect of Proline Analogues on Activity of Human Prolyl Hydroxylase and the Regulation of HIF Signal Transduction Pathway

**DOI:** 10.1371/journal.pone.0095692

**Published:** 2014-04-22

**Authors:** Xiaoyan Ma, Xiaoxin Wang, Jing Cao, Zhirong Geng, Zhilin Wang

**Affiliations:** State Key Laboratory of Coordination Chemistry, School of Chemistry and Chemical Engineering, Nanjing University, Nanjing, Jiangsu, PR China; Johns Hopkins School of Medicine, United States of America

## Abstract

Hypoxia inducible factor 1 (HIF-1) plays a pivotal role in cellular responses to hypoxia. Prolyl hydroxylase 3 (PHD3) degrades HIF-1α under normoxic conditions through the hydroxylation of HIF-1α for proteolysis. Inhibiting PHD3 activity is crucial for up-regulating HIF-1α, thereby acting as a potential target for treating hypoxia-related diseases. In this study, two proline analogues (PA1 and PA2) were screened as PHD3 inhibitors with apparent EC_50_ values of 1.53 and 3.17 µM respectively, indicating good inhibition potency. Nine proteins, significantly regulated by PA1, were identified using 2-DE coupled with MALDI-TOF/TOF MS. Pyruvate kinase isozymes M1/M2 (PKM) and alpha-enolase 1 (ENO1), which are key modulators of glycolysis, are directly regulated by HIF-1α. Moreover, VEGF, a signal protein stimulating angiogenesis, was strongly promoted by PA1. Our findings suggest that PA1 stabilized HIF-1α as well as up-regulated glycolysis and angiogenesis proteins. Herein, for the first time, we systematically studied proline analogue PA1 as a PHD3 inhibitor, which provides innovative evidence for the treatment of HIF-related diseases.

## Introduction

Oxygen homeostasis, an important organizing principle for the development and physiology of living organisms, can be disrupted by cardiovascular, pulmonary, hematological diseases and cancers [1], [2]. Hypoxia inducible factor 1 (HIF-1), which functions as a master regulator of oxygen homeostasis [3],[4], is a heterodimer consisting of an alpha subunit (HIF-1α) and a beta subunit (HIF-1β). Under normoxic conditions, cellular HIF-1α is regulated by hydroxylation with prolyl hydroxylase 3 (PHD3), ubiquitination, and proteasomal degradation. Under hypoxic conditions, PHD3-modified HIF-1α is greatly decreased, resulting in its stabilization and accumulation [5]–[8]. Because PHD3 is the key enzyme regulating HIF activity in response to pO_2_, inhibiting PHD3 is an attractive target for pharmaceuticals to treat diseases related to HIF up-regulation, such as myocardial infarction, stroke, and anemia, among others [9]–[11].

PHD3 belongs to the 2-oxoglutarate dependent dioxygenase superfamily, which requires oxygen, iron, 2-oxoglutarate, and ascorbate for the hydroxylation reaction. Therefore, PHD3 may be inhibited by depletion of or competition for these factors that stabilize HIF-1α [12]–[15]. Three predominant types of small molecules have been reported to inhibit PHD activity, i.e. metal ions [16],[17], iron chelators [18]–[22], and proline analogues [23]–[25]. Metal ions such as cobalt can inactivate the enzymes by occupying an iron-binding site on proline hydroxylases [5]. Most iron chelators are 2-oxoglutarate (2OG) analogues which share similar simple 2OG scaffolds to chelate iron in a bidentate fashion [20]. Some classical chelators, such as N-oxalylglycine (NOG) [26], dimethyloxalylglycine (DMOG) [27], ethyl-3, 4-dihydroxybenzoate (3, 4-DHB) [12], and deferoxamine mesylate (DFO) [12], can competitively inhibit the activity of PHD and stabilize HIF-1α to repair chronic disease anemia and neuronal injury. However, iron chelators cannot exclusively bind to PHDs and may disturb other iron-containing proteins that maintain normal physiology and biochemistry [28]. Still, these reports raise new questions as to the selectivity of HIF hydroxylase inhibitors and the extent to which their biological activity is mediated solely by inhibition of PHDs and FIH. PHD3 hydroxylates proline residue 564 on the HIF-1α oxygen-dependent degradation domain for proteasomal destruction [7],[29]. Ahn synthesized peptides containing 556–575 residues of HIF-1α with modifications at the Pro-564 to act as proline analogues and reported that they specifically inhibit PHD2 [30]. Thus, proline analogues can specifically inhibit HIF hydroxylase activity.

Hypoxia is one of the most potent inducers of gene expression, especially genes involved in glycolysis to maintain cellular energy [31],[32]. HIF plays an important role in cellular response by regulating downstream genes associated with glycolysis, angiogenesis, and metastasis [33]. HIF-targeted glucose metabolism genes include glucose transporter-1, 3 (GLUT-1, 3) [34], enolase-1 (ENO1) [35], lactate dehydrogenase-A (LDHA) [35], 6-phosph-ofructo-2-kinase/fructose-2, 6- bisphosphate-3 (PFKFB3) [36], and pyruvate kinase M (PKM) [37]. Many PHD inhibitors adapt to hypoxia by stabilizing HIF and up-regulating GLUT-1, 3 [38],[39]. PKM is a key enzyme in glucose metabolism [40]. However, proline analogues, which promote PKM via stabilization of HIF-1α as an inhibitor of PHD3, have been studied little and they have not been extensively studied *in vivo*.

**Figure 1:**
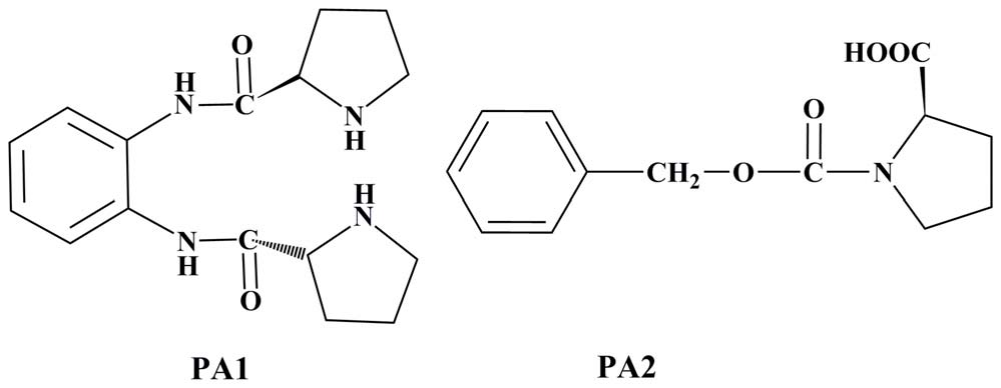
Structures of PA1 and PA2.

To date, proline analogues that have been reported to inhibit PHD activity are peptide ones. Most peptide analogues contain 556–575 residues of HIF-1α with modifications at the Pro-564 [24],[25],[30]. Under normoxic conditions, proline residue 564 on the HIF-1α oxygen-dependent degradation domain is hydroxylated by PHDs [6]. The sequences of 556–575 residues of HIF-1α are DLDLEMLAPYIPMDDDFQL, and the neighboring amino acid of proline residue 564 is tyrosine [30]. Thus, we designed and synthesized two proline analogues (PA1 and PA2, Figure 1) containing benzene ring like tyrosine. Structure of PA1 resembles PYIP peptide because two proline residues are connected by o-phenylenediamine. We explored the PA1 and PA2 effects on PHD3 activity and found PA1 was a competitive inhibitor for the substrate. Furthermore, the regulatory effect of PA1 on the signal transduction pathway in HIF-glycolytic metabolism in NCI-446 cells was studied using RT-PCR, Western-blot and 2-DE coupled with MALDI-TOF/TOF MS methods. PA1 inhibited PHD3 activity, promoted HIF-1α accumulation, and up-regulated HIF-1α target genes *PKM* and *ENO1* as well as protein VEGF. PA1 likely regulates the special signal transduction pathway within HIF-glycolytic metabolism as a potential PHD inhibitor.

## Materials and Methods

### Materials

Expression host, *E. coli* BL21 (DE3) pLysS, and pET32α (+) vectors were acquired from Novagen. Isopropyl β-D-thiogalacto-pyranoside (IPTG), dithiothreitol (DTT), 2-oxoglutarate, ascorbate, bovine serum albumin (BSA), and catalase were purchased from Sigma. HIF-1α peptide corresponding to residues 556–574 (DLDLEMLAPYIPMDDD-FQL) was synthesized by Shanghai Apeptide Co., Ltd. BCA Protein Assay Kit was obtained from KeyGEN BioTECH. All other reagents were of analytical grade and all solutions were prepared using Milli-Q deionized water. PA1 and PA2 were synthesized as reported by Weeks and Berger [41],[42], respectively.

### Protein Expression and Purification

Recombinant human PHD3 enzyme was expressed in *E. coli* as described previously [43],[44]. The cultured cells were centrifuged at 10,000 rpm for 10 min at 4°C, and each gram of cell pellet was resuspended in 5 mL of binding buffer (20 mM Na_2_HPO_4_, 2 M NaCl, 5 mM imidazole, pH 7.0), followed by sonication on ice and centrifugation at 14,000 rpm for 10 min. The post-centrifugation supernatant was filtered through a 0.45-µm hydrophilic polypropylene membrane to prevent clogging of the chromatography medium. HisTrap FF crude column was pre-equilibrated with 3 column volumes of binding buffer, to which the supernatant was applied, and the flow-through was collected for analysis. The column was then washed with 5 column volumes of binding buffer and then 10 column volumes of washing buffer containing 100 mM imidazole (otherwise identical to binding buffer). When the absorption was back to baseline, target proteins were eluted with a linear gradient of imidazole from 100 to 500 mM for 7 column volumes at 3 mL/min. The elution was continued with 500 mM imidazole for 2 column volumes to ensure bound proteins were all eluted. Collected fractions were concentrated to less than 2 mL by ultrafiltration. Gel filtration was performed on Superdex 200 column equilibrated with buffer GF (20 mM Na_2_HPO_4_, 0.5 M NaCl, 1 mM DTT, pH 6.5). A portion of 2 ml of the concentrated Ni-NTA fraction was applied to the column and eluted with buffer GF. The flow rate was 0.8 ml/min for optimal separation. Fractions containing PHD3 were collected and concentrated. Protein purity was verified by SDS-PAGE and MALDI-TOF/MS. Protein concentration was measured by using the BCA Protein Assay Kit.

### Activity Assay

PHD3 hydroxylation activity was measured via a fluorescence-based method developed by McNeill [45], and based on reported derivatization methods for 2OG using o-phenylenediamine (OPD) to yield a fluorescent derivative [46]. The PHD3 activity assay was carried out by mixing 2 mg/mL BSA, 1 mM DTT, 2 mM ascorbate, 0.6 mg/mL catalase, 50 µM FeCl_2_ (prepared as 500 mM stock in 20 mM HCl and diluted with water), HIF 19 peptide, enzyme PHD3 and 20 mM (pH 7.0) PBS to a final volume of 96 µL on an ice bath. The reaction was initiated by adding 4 µL of 2OG (160 µM) to the reaction mixture. The concentration of remaining 2OG was quantified after the catalytic reaction was carried out by detecting the fluorescent intensity of the derivatization product between 2OG and OPD on a Perkin-Elmer spectrometer (excitation = 340 nm; emission = 420 nm). The structures of PA1 and PA2 in the reaction mixture were detected by NMR spectra using Bruker AVANCE III 600 MHz.

### Cell Culture

Human small cell lung cancer NCI-H446 cells [47],[48], was obtained from the Cell Bank of the Chinese Academy of Sciences (Shanghai, China) and cultured in Dulbecco’s modified Eagle’s medium with 10% heat-inactivated fetal bovine serum, 100 units/mL penicillin and 100 units/mL streptomycin in a humidified 5% CO_2_ environment at 37°C. At 70%–80% confluence, cells were harvested after treatment with 0.25% (w/v) trypsin-EDTA, and were then reseeded for expansion.

### Cell Proliferation Assay

3-(4, 5-Dimethylthiazol-2-yl)-2, 5-diphenylte-trazolium bromide (MTT) assays were applied to test cell viability. Briefly, 2×10^4^ NCI-H446 cells were seeded onto 96-well plates and incubated for 12 h. Then the medium was changed to one containing various concentrations of PA1, DFO and CoCl_2_. Subsequently, a fraction of 20 µL MTT (0.5 mg/mL final concentration) was added to each well. After incubation for 4 h, 150 µL of DMSO was added to dissolve the crystals. Absorbances at 570 nm were monitored by an automatic ELISA plate reader.

### Two-dimensional Electrophoresis (2-DE)

PA1-treated NCI-H446 cells were counted and harvested by centrifugation, washed twice with Tris-buffered sucrose (10 mM Tris base, 250 mM sucrose, pH 7.0), and dissolved in lysis buffer containing 7 M urea, 2 M thiourea, 4% CHAPS, 50 mM DTT, complete mini protease inhibitor cocktail tablet and 0.2% pH 3–10 IPG buffer. After incubation on ice for 1 h, the samples were centrifuged at 14,000 × g for 30 min at 4°C. The supernatant was quantified using a 2-D Quant Kit. Protein (1 mg) was diluted in rehydration buffer containing 7 M urea, 2 M thiourea, 2% CHAPS, 25 mM DTT, 0.2% pH 3–10 IPG buffer and 0.002% bromophenol blue, and was applied to 24 cm IPG strips with a linear range of pH 3–10. The first dimension was carried out on a Multiphor II IEF system. IEF was performed at 20°C under the following conditions: 100 V for 2 h, 250 V for 30 min, 500 V for 30 min, 1,000 V for 1 h, 10,000 V for 2 h and 10,000 V for 60,000 V.h. After IEF, the IPG strips were equilibrated for 15 min in a buffer containing 6 M urea, 20% glycerol, and 2% SDS (in 0.05 M Tris-HCl buffer, pH 8.8) with 2% DTT, and then equilibrated for another 15 min in the same buffer but with 2.5% iodoacetamide substituting for DTT. Then 2D separations were carried out on 12.5% SDS-polyacrylamide gels at 2 w per gel for 40 min and then at 15 w per gel using an Ettan DALTsix vertical electrophoresis system until the dye front reached the gel bottom. Gels were visualized by Coomassie staining to compare spot patterns between controls and PA1-treated cells. Gels were scanned using Epson Expression 10000 XL, and the image was analyzed with Image-Master 2D software. The spot differential was defined as change in spot %volume upon comparison of average gels between treated and control groups.

### Mass Spectrometry and Protein Identification

Differentially expressed protein spots on 2-DE gels, which were stained using Coomassie brilliant blue G250 dye, were identified for subsequent mass spectrometry. Selected spots had a greater than 2-folds change as evidenced by image analysis. After washing twice with Milli-Q water, gel pieces were washed in 25 mM ammonium bicarbonate/5% ACN, and twice in 25 mM ammonium bicarbonate/50% ACN. Gel pieces were dried in 100% ACN and digested in 10 µL of trypsin (10 ng/µL, Trypsin Gold, mass spectrometry grade) in 25 mM ammonium bicarbonate at 37°C overnight. Peptide digestion (1 µl) was mixed with the matrix solution (10 mg α-cyano-4-hydroxycinnamic acid in 1 ml of 50% acetonitrile and 0.1% trifluoroacetic acid), and 1 µl of this mixture was applied to the stainless steel plate of the mass spectrometer. Mass spectrometry was performed using a MALDI-TOF/TOF MS. Before real sample acquisition, six calibrated spots were used to optimize signals and parameters. Proteins were then identified by searching the Swiss-Prot database and NCBI nr protein using the MASCOT search engine of Matrix Science.

### Western Blotting

NCI-H446 cells were harvested 24 h after treated the three doses of PA1, DFO and CoCl_2_ and lysed using lysis buffer containing 20 mM Tris-HCl (pH 8.0), 250 mM NaCl, 0.4 mM Na_3_VO_4_, 1% SDS and complete mini protease inhibitor cocktail tablet. Samples were separated by 12% SDS-PAGE and transferred to Immobilon-P transfer membrane. Membranes were blocked with 5% nonfat milk in TBS containing 0.1% Tween-20 at room temperature for 1 h, and then incubated with anti-β-Actin (1∶2,000) or anti-HIF1α (1∶1,000). Antibodies were diluted in TBS with 5% nonfat milk at 4°C overnight. Blots were incubated with HRP-conjugated anti-rabbit secondary antibody (1∶5,000) and anti-mouse secondary antibody (1∶5,000) for 1 h, and detected by using ECL. Blot images were scanned and analyzed using Quantity One software.

### Semi-quantitative Reverse Transcription-PCR analysis

Total RNA was isolated from PA1-treated NCI-H446 cells using Trizol reagent following the manufacturer’s instructions. RNA was quantified using Nanodrop ND-1000. Two micrograms of each RNA sample was reverse-transcribed into cDNA using a primeScript RT-PCR kit. PCR was conducted using cDNA as the template and TakaRa Taq kit. The amplified product was detected by 1% agarose gel electrophoresis, scanned and analyzed using Quantity One software. Nucleotide sequences of the primers are shown in Table 1.

**Table 1:** Primers used for reverse transcriptase-polymerase chain reaction.

Primer	Nucleotide sequence(5′-3′)	Amplicon size
EIF5A sense	CGTAAGAATGGCTTTGTGGT	238
EIF5A antisense	TGTCCTGGAGCAGTGATAGGT	
PSME2 sense	CCACCCAAGGATGATGAGAT	257
PSME2 antisense	ACTTTGGTCTTGACGGCATT	
HNRNPC sense	TTGCCTTCGTTCAGTATGTT	378
HNRNPC antisense	CACTCTTAGAATTGAAGCCACT	
MAT2A sense	TACAATCTACCACCTACAGCC	240
MAT2A antisense	GAGACCTGAACAAGAACCCT	
ENO1 sense	CGCATTGGAGCAGAGGTTTA	281
ENO1 antisense	AGCTGGTCAGGCGAGATGTA	
HSPA8 sense	CGCCTTTACGGACACTGAAC	353
HSPA8 antisense	CTTTGGTAGCCTGACGCTGA	
PMPCB sense	GCTCATCTCAATGCCTATACCT	373
PMPCB antisense	ATTCATCATGGGAAACACCT	
PKM sense	AATCACGCTGGATAACGCCTAC	322
PKM antisense	TGCCTTGCGGATGAATGACG	
HNRNPH2 sense	TAGCCCTGATACTGCCAACG	230
HNRNPH2 antisense	ACCTGTGCCCTATTCTTTCC	
ACTIN sense	GACCTGACTGACTACCTC	430
ACTIN antisense	TCTTCATTGTGCTGGGTGC	

### Statistical Analysis

Data are representative of three or more independent experiments. The student’s t-test was used for statistical analysis and P<0.05 was considered significant.

## Results

### Inhibited Activity of Recombinant Human PHD3 by PA1 and PA2

The cloning, expression, and Ni-NTA purification of recombinant human PHD3 were carried out as described previously [49]. Gel filtration was performed after Ni-NTA to further purify the enzyme to eliminate remaining impurities and imidazole. Reducing agents stabilized PHD3 throughout gel filtration. Approximately 90% of high-purity PHD3 was eluted with molecular masses of ∼45 kDa. Proline of the HIF-1α peptide formed hydroxyproline *in vitro* by hydroxylation with recombinant human PHD3.

**Figure 2:**
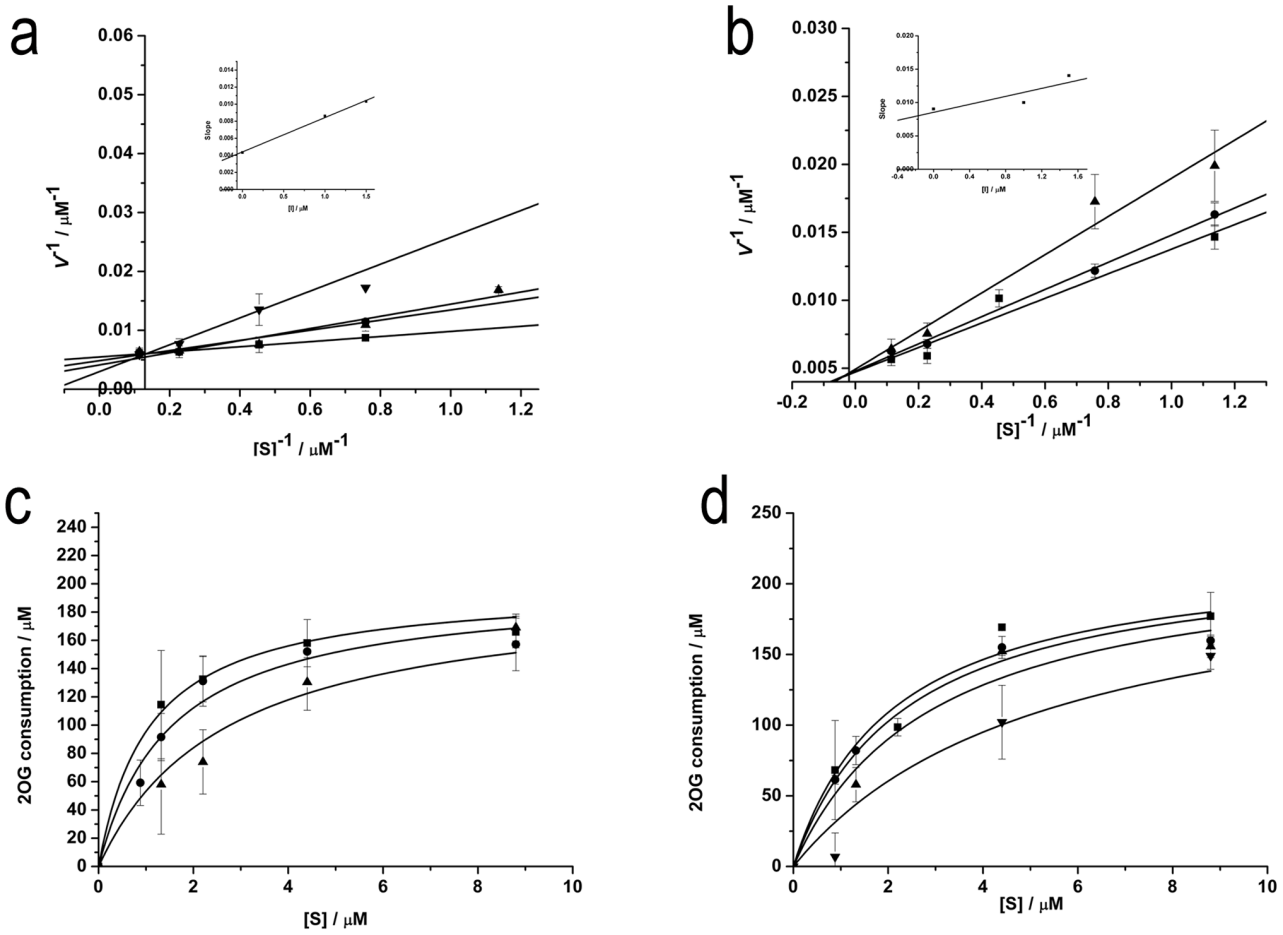
Enzyme kinetics of HIF PHD3. a b Double reciprocal plots of the relationship between the concentration of peptide substrate and initial velocity. Enzyme concentration was 5 µg. a Plots for PA1 at 0 µM (▪), 1 µM (•), 1.5 µM (▴) and 2 µM (▾). b Plots for PA2 at 0 µM (▪), 1 µM (•) and 1.5 µM (▴). c d Concentration dependence of inhibition of PHD3 activity by PA1 and PA2. Data were analyzed by global fitting of the Michaelis–Menten equation. c Plots for PA1 at 0 µM (▪), 1.5 µM (•) and 2 µM (▴). d Plots for PA2 at 0 µM (▪), 1 µM (•), 1.5 µM (▴) and 3 µM (▾). Data are presented as mean ± S.D. of three independent experiments.

**Figure 3:**
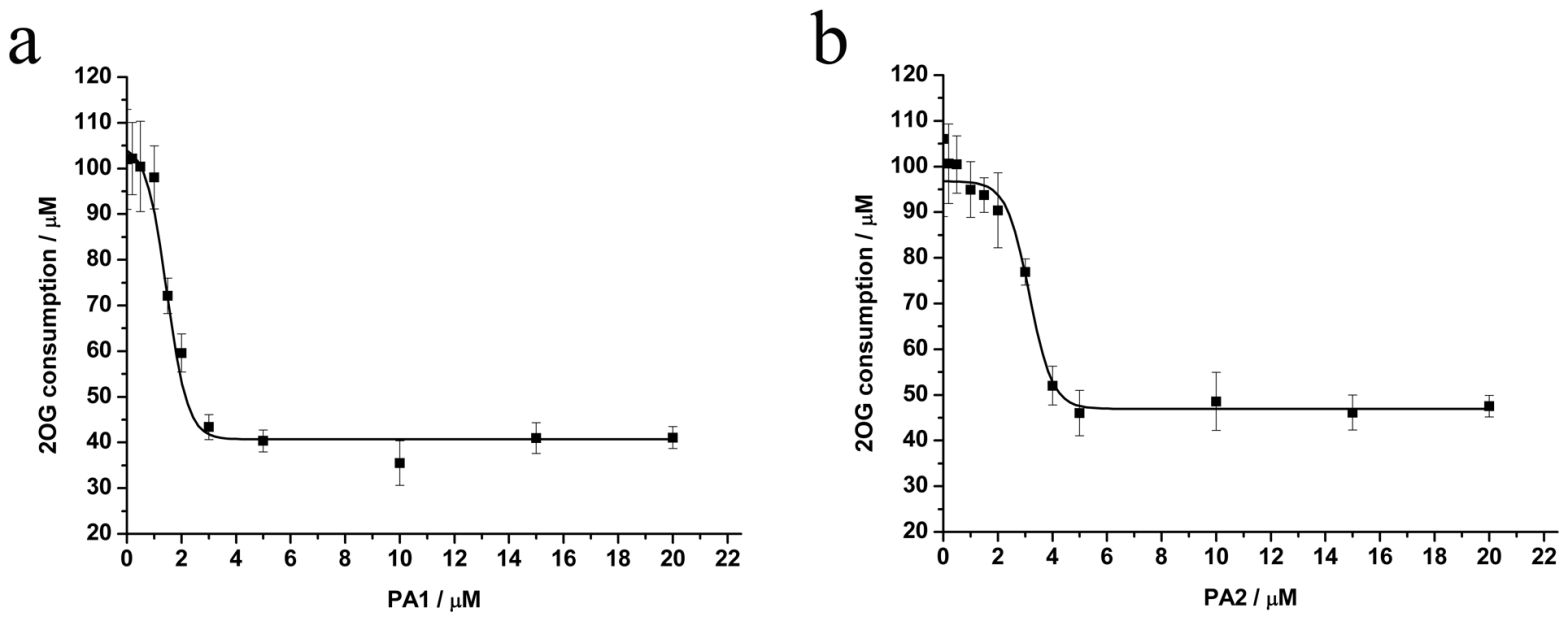
Effects of a PA1 and b PA2 on hydroxylation of PHD3. Incubations were carried out at the KM of HIF-1α peptide substrate and a saturating concentration of 2OG, with increasing compound concentration (0–20 µM). EC_50_ values were estimated by dose regression of initial velocity versus compound concentration. Data are presented as mean ± S.D. of three independent experiments.

**Figure 4:**
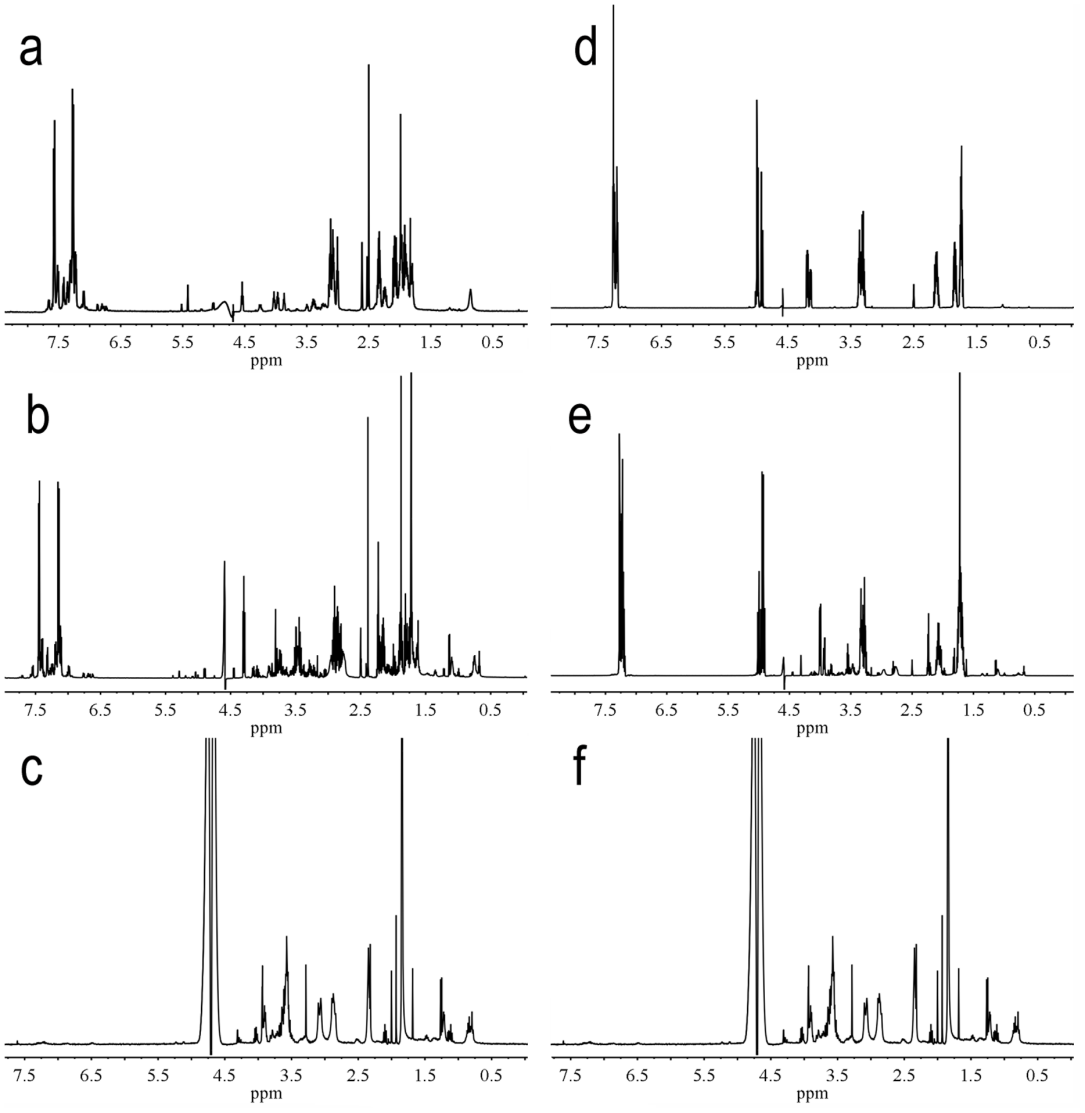
NMR spectra of PA1 and b PA2. **a, d** PA1, PA2 in D_2_O. **b, e** PA1, PA2 in reaction mixture. **c, f** Reaction mixture (100 µL, containing 14 µg PHD3, 1.0 mM DTT, 2.0 mM ascorbate, 50 µM FeSO4, 0.16 mM 2-oxoglutarate, 2.0 mg/mL BSA, 0.60 mg/mL catalase, and 66.55 µM HIF-1α peptide in 20 mM phosphate buffer (pH 7.0)).

Kinetic analysis characterized recombinant human PHD3 inhibition by PA1 and PA2 with 10^−6^–10^−5^ M HIF-1α peptide. The Michaelis-Menten equation provided *Km* of (2.03±0.67) µM. Double-reciprocal (1/*V* versus 1*/*[HIF-1α peptide]) plots showed inhibition types of the proline analogues. PA1 was inclined to be competitive, whereas PA2 was non-competitive inhibitors for the substrate. *K_i_* values were 1.09 and 2.38 µM for PA1 and PA2 respectively (Figure 2a, b), suggesting tighter binding of the competitive inhibitors to the enzyme catalytic site. The two compounds inhibited enzyme catalytic activity in a concentration-dependent manner (Figure 2c, d), with apparent EC_50_ values for PA1 and PA2 of 1.53 and 3.17 µM, respectively (Figure 3a, b). The EC_50_ value of PA2 was slightly greater than that of PA1, so PA1 was chosen for the following cell studies. NMR experiments showed the structures of PA1 (Figure 4a, b, c) and PA2 (Figure 4d, e, f) were retained in the in vitro enzymatic assay.

### Slight Decrease in NCI-H446 Cell Viability by PA1

**Figure 5:**
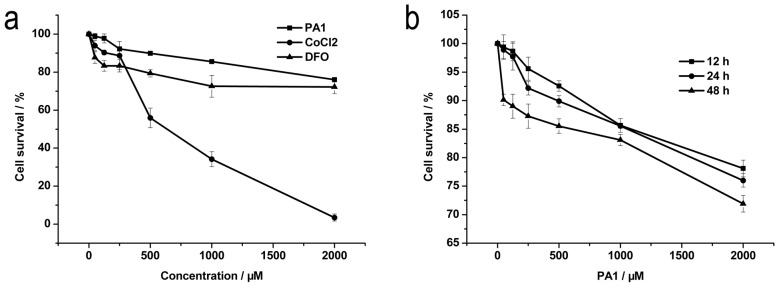
Cytotoxicity of PA1 on NCI-H446 cells measured with MTT. **a** PA1 (12 h (▪), 24 h (•), 48 h (▴)). **b** 24 h (PA1 (▪), CoCl_2_ (•), DFO (▴)). Data are presented as mean ± S.D. of three independent experiments.

Cytotoxicity of PA1 on NCI-H446 cells was assessed by MTT tests after 12, 24 and 48 h of treatment with increasing PA1 concentrations (Figure 5a). PA1 suppressed the proliferation of NCI- H446 cells in a time- and dose-dependent manner, but inhibition was mild even after 48 h treatment of 2,000 µM PA1 (cell viability: >72%). In the presence of 1,000 µM PA1, survival of NCI-H446 cells increased to 85 and 83% after 24 and 48 h, respectively. Hence, 1,000 µM PA1 was used thereafter. DFO and CoCl_2_ that are both well-known PHD inhibitors are used as positive controls. Figure 5b showed the cytotoxicity effect of PA1 in comparison with DFO and CoCl_2_ is lowest, suggesting the good biocompatibility of PA1. Accordingly, 1000 µM PA1 used within cells thereafter was feasible.

### HIF-1α Expression is Promoted by PA1 Under Normoxic Conditions

**Figure 6:**
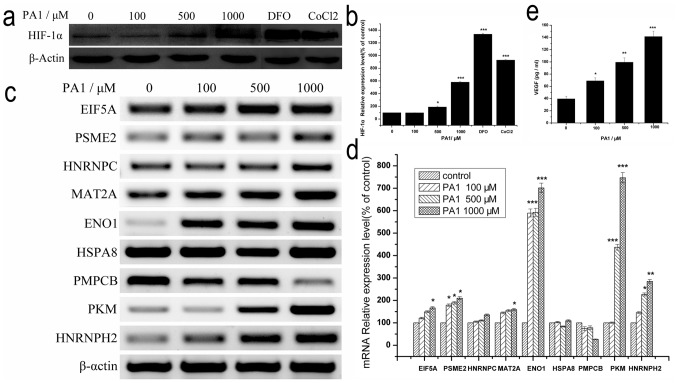
Expression of protein and mRNA in NCI-H446 cells treated with different concentrations of PA1 under normoxic conditions. **a** HIF-1α protein in H446 cells measured by Western blot, 100 µM DFO and 100 µM CoCl_2_ were used as positive controls. **b** Measurement of stabilized HIF-1α protein. **c** Pre-validation of proteomic mRNA analysis in control and PA1-treated NCI-H446 cells. To measure the degree of change in identified proteins, we measured mRNA in PA1-treated NCI-H446 cells. mRNA expression of seven proteins were measured using RT-PCR. **d** mRNA expression of seven proteins in NCI-H446 cells treated with different concentrations of PA1 under normoxic conditions. **e** ELISA was used to measure secreted VEGF protein. Data are presented as mean ± S.D. of three independent experiments. (*P<0.05, **P<0.01, ***P<0.001 vs. non-treated control group).

To verify the HIF-1α pathway, HIF-1α expression in NCI-H446 cells treated with PA1 under normoxic conditions was detected by Western blot, treating cells with PA1 for 24 h up-regulated HIF-1α protein in a dose-dependent manner (Figure 6a). Particularly, HIF-1α protein was increased six-fold in response to 1,000 µM PA1 (Figure 6b). Well-known PHD3 inhibitors DFO and CoCl_2_ were used as positive controls. Treating cells with 100 µM DFO for 24 h increased HIF-1α protein almost thirteen-folds, and 100 µM CoCl_2_ increased HIF-1α protein approximately nine-folds. Compared with the positive control, the potency of PA1 increasing HIF-1α protein was half that of DFO and three quarters that of CoCl_2_. Ergo, PA1 was a fine PHD3 inhibitor to up-regulate HIF-1α protein.

### Identification of Differentially Expressed Protein Spots with 2-DE/MALDI-TOF MS

**Figure 7:**
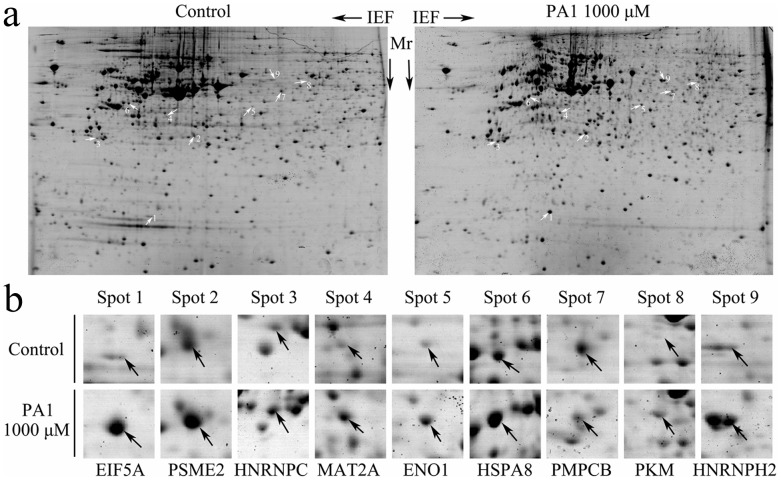
Protein expression patterns of control and PA1-treated NCI-H446 cells. **a** After a 24-h treatment of cells with 1,000 µM PA1, total protein of NCI-H446 cells was extracted, and then separated by pI and molecular weight. Both panels show a 2-DE gel that was CBB-stained for protein visualization. Differentially expressed protein spots from control and PA1-treated groups are indicated by arrows. Proteins were then identified using MALDI-TOF/TOF MS (Table 2). **b** Gel regions in which spots correspond to differentially expressed proteins are shown.

To identify proteins associated with PA1-induced HIF-1α stabilization, protein expression patterns of control and PA1-treated groups were compared using 2-DE proteomic analysis (Figure 7a). Using Image-Master 2D software, more than 600 protein spots were detected. Spot densities were measured with normalization based on total spot volumes on the gel. Nine spots in control and PA1-treated group were selected (Figure 7b) and 9 well-resolved and matched spots among six gels were chosen to calculate the spot density deviations (Table 2). Protein spots were cut out, in-gel digested, and analyzed by MALDI-TOF/TOF MS. Database searching for MS fingerprint profiles indicated that differentially expressed proteins had various functions, including metabolism (alpha-enolase, pyruvate kinase isozymes M1/M2, S-adenosylmethionine synthase isoform type-2, eukaryotic translation initiation factor 5A-1, and mitochondrial-processing peptidase subunit beta), regulation of gene expression (heterogeneous nuclear ribonucleoprotein H2, heterogeneous nuclear ribonucleoproteins C1/C2, and heat shock cognate 71 kDa protein), and roles in the cell cycle (proteasome activator complex subunit 2).

**Table 2:** Identification of differently expressed proteins spots in the control and PA1-treated groups.

No^a^	AccessionNumber^b^	Proteinname	Gene name	Quantitativechanges (%)^c^	TheoreticalMW(Da)/pI	pathway
1	P63241	Eukaryotic translation initiation factor 5A-1	EIF5A	673.5±25.5	17.049/5.08	Metabolism of proteins
2	Q9UL46	Proteasome activator complex subunit 2	PSME2	512.6±15.0	27.555/5.54	Signal Transduction
3	P07910	Heterogeneous nuclear ribonucleoproteins C1/C2	HNRNPC	275.7±16.0	33.707/4.95	Gene Expression
4	P31153	S-adenosylmethionine synthase isoform type-2	MAT2A	236.4±8.9	43.975/6.02	Metabolism
5	P06733	Alpha-enolase	ENO1	259.3±17.2	47.481/7.01	Metabolism, HIF-1α transcriptionfactor network
6	P11142	Heat shock cognate 71 kDa protein	HSPA8	215.2±7.5	7.082/5.37	Gene Expression
7	O75439	Mitochondrial-processing peptidase subunit beta	PMPCB	−249.4±8.2	55.073/6.38	Metabolism of proteins
8	P14618	Pyruvate kinase isozymes M1/M2	PKM	208.6±4.5	58.470/7.96	Metabolism, HIF-1α transcriptionfactor network
9	P55795	Heterogeneous nuclear ribonucleoprotein H2	HNRNPH2	426.7±8.3	49.517/5.89	Gene Expression

a Spot number on 2-DE gel (Fig. 7).

b Swiss-Prot accession numbers.

c The values show the percent change of proteins. −: down-regulated, +: up-regulated (n = 3).

### HIF-1α Pathway

More than 40 hypoxia-inducible-factor target genes have been characterized by the functional delineation of HIF binding to hypoxia-response elements in transcriptional control sequences. Thirteen differentially expressed proteins were identified in control and PA1-treated groups, and two proteins (alpha-enolase and pyruvate kinase isozymes M1/M2) were directly related to the HIF-1-α transcription factor network.

### Preliminary Validation of Identified Proteins by RT-PCR

RT-PCR analysis was used as an alternative method to detect differentially expressed proteins (Figure 6c). PA1 induced mRNA expressions of nine genes in a concentration-dependent manner to different extents (Figure 6d). The two HIF-1α related genes, ENO1 and PKM, were significantly up-regulated and 1,000 µM PA1 up-regulated PKM seven-folds at the mRNA level.

### PA1-promoted Expression of VEGF

Although HIF-1 is a powerful modulator of many hypoxia-related proteins, VEGF is the most well-known target for HIF-1 activation [50]. Accordingly, VEGF was utilized to investigate the correlation between PA1 and HIF-1 expression. VEGF secretion was measured in cell culture supernatants with an ELISA kit. PA1, especially 1,000 µM PA1, significantly increased VEGF secretion compared with control (Figure 6e).

## Discussion

Oxygen disorder diseases represent very real burdens to human health and include heart, cerebrovascular, and chronic obstructive lung disease. These conditions demand effective treatment protocols that save lives and decrease healthcare costs. HIF-1 is an important factor for regulating cellular and systemic oxygen homeostasis and HIF-1 is regulated through the hydroxylation of the HIF-1α proline residue by PHD. Therefore, developing novel pharmaceuticals to inhibit PHD activity may offer treatment strategies for these diseases. Compared with metal ions and chelators, proline analogues have been studied very little, so to address this deficit in the literature and explore the effects of proline analogues on PHD3 activity and HIF signaling, we performed proteomic analysis using 2-DE methods.

Prior to proteomic analysis, proline analogue inhibition of and affinity for recombinant human PHD3 were measured. PA1 competitively inhibited catalytic activity in a concentration-dependent manner. Iron binding assay with PA1 and PA2 indicated that PA1 and PA2 were not iron chelators. Low EC_50_ values suggested that these two compounds had moderate inhibitory activities compared with polynitrogen compounds [43] and macrocyclic polyamines [49] studied previously, which could be attributed to binding to iron and inactivation of hydroxylation via the formation of stable coordination complexes. However, PA1 competed for the HIF-1α substrate proline site, thus decreasing hydroxylation activity. This implies that proline analogues PA1 and PA2 are potential inhibitors *in vitro* and these data motivate us to explore the effects *in vivo*.

A major advantage of 2-DE and MS technologies is that they offer insight into the understanding variations for treatment with the potential inhibitor PA1. Differential proteins were identified using 2-DE-based-proteomic analysis, and nine differentially expressed protein spots were identified using MALDI-TOF/TOF MS, among which ENO1 and PKM genes were directly related with HIF-1α and glucose/energy metabolism.

In this study, PA1 increased HIF-1α. HIF-1 expression activates transcription of genes encoding proteins that mediate angiogenesis, metastasis, and a shift from oxidative to glycolytic metabolism [51],[52]. These target genes are involved in many ischemia oxygen deficiency diseases such as pulmonary arterial hypertension, myocardial infarction, stroke, precursors to childhood epilepsy and almost all tumor types [53],[54]. Thus, researchers studied HIF expression to investigate the up-regulation of angiogenesis and glycolytic metabolism [55]–[57]. Glycolysis is stimulated by hypoxia or cerebral ischemia, or both, as the body tries to maintain optimal cellular energy balance despite oxygen debt. It has been shown that enzymes of glycolysis tend to be regulated in hypoxia [58],[59] and ischemia [60]. PKM is a key enzyme that determines glycolytic activity and functions as a coactivator to stimulate HIF-1 transactivation of target genes encoding GLUT1, LDHA, and PDK1 to increase glucose uptake and inhibit O_2_ consumption [40],[61]. Pyruvate kinase activity was reported to increase in hypoxia and/or ischemia [58],[59],[62],[63]. So far, little is reported about compounds that augment PKM via HIF stabilization. We found that PA1 increased PKM significantly. Moreover, the gene for ENO1 was up-regulated by PA1. ENO1, a multifunctional enzyme, participates in growth control and hypoxia tolerance [35]. ENO1 expression is significantly upregulated during cellular growth [64] and in hypoxic situations, it acts as a stress protein that is up-regulated via activation of HIF-1 [65] and is postulated to provide protection to cells by increasing anaerobic metabolism [66]–[68]. Kobayashi and colleagues reported that the introduction of α-enolase appeared to increase cell survival and cardiomyocyte contractility more than expected from maintenance of ATP levels alone. These findings suggest that α-enolase is essential for protecting the heart from ischemic injury [69]. Hence, PA1 enhanced glycolytic metabolism by targeting PKM and ENO1 and PA1 induced VEGF expression in NCI-H446 cells. Although many genes associated with hypoxia are expressed via this pathway, VEGF is the typical HIF-1-modulated factor and an HIF-1 activation indicator [70]. Our data provide further evidence regarding PA1-induced HIF-1 activation. The expression of VEGF by HIF activation facilitated angiogenic responses [71],[72], and based on up-regulated PKM, ENO1 and VEGF by PA1, PA1 might mitigate HIF-related diseases.

## Conclusion

We systematically studied a proline analogue PA1 as a PHD3 inhibitor *in vitro* and investigated its effect on the HIF signal pathway in cellular systems. PA1 enhanced glycolytic metabolism and angiogenesis by activating HIF-1α and modulating HIF-1α-related genes for PKM and ENO1 and protein VEGF. The results provide evidence for the design of eligible and effective pharmaceutical agents for hypoxia-related diseases.
